# Social context surrounding HIV diagnosis and construction of masculinity: a qualitative study of stigma experiences of heterosexual HIV positive men in southwest Nigeria

**DOI:** 10.1186/s12889-016-3165-z

**Published:** 2016-06-13

**Authors:** Titilayo Ainegbesua Okoror, Catherine Olufunke Falade, Ebunlomo Mary Walker, Adetayo Olorunlana, Agaptus Anaele

**Affiliations:** Department of Africana Studies, Binghamton University, State University of New York, 4400 Vestal Parkway East, PO Box 6000, Binghamton, NY 13902-6000 USA; Department of Pharmacology & Therapeutics, College of Medicine, University College Hospital, Univeristy of Ibadan, Queen Elizabeth Road, Private Mail Bag 5017, Ibadan, Oyo Nigeria; Director, Initiative for Integrated Community Welfare in Nigeria (IICWIN), 8 Alfonso Road, Shasha/Ojoo, Ibadan, Secretariat P.O. Box 29802, Oyo Nigeria; Department of Sociology, Faculty of Social Science, University of Ibadan, Ibadan, P.O. Box 0234, Oyo Nigeria; Department of Marketing Communication, Emerson College, 120 Boylston Street, P.O. Box 0234, Boston, MA 02116-4624 USA

**Keywords:** HIV stigma, Heterosexual HIV positive men, Nigeria, Masculinity, Culture

## Abstract

**Background:**

Though research has documented experiences of stigma and its effects on the lives of women living with HIV/AIDS, there is limited research on heterosexual positive HIV men experience of stigma in Nigeria. This study explored how social context surrounding HIV diagnosis impacts stigma experiences of heterosexual HIV positive men and their construction of masculinity in southwest Nigeria.

**Methods:**

Using purposive sampling, 17 heterosexual HIV positive men were recruited through community based organization to participate in two hours focus group discussions or 45 min in-depth interviews that were audio-recorded. Without using the word stigma, discussions and interviews were guided by four questions that explored participants’ experiences of living with HIV/AIDS. Interviews and discussions were conducted in three languages: English, Yoruba and Pidgin English. Thematic data analysis approach was in coding transcribed data, while social constructivist thinking guided data analysis.

**Results:**

Participants ranged in age from 30 to 57 years old, and all were receiving antiretroviral therapy. Findings indicated that participants’ experiences of stigma might be moderated by the social context surrounding their HIV diagnosis, and whether they have met the socio-cultural construction of masculinity. Participants whose diagnosis were preceded by immediate family members’ diagnosis were less likely to report experiencing HIV stigma and more likely to report “not feeling less than a man” and educating others about HIV/AIDS. Contrarily, participants whose diagnosis was preceded by their own sickness were more likely to report isolation, sigma and feeling of being less than a man. All participants reported limiting their sexual intimacy, and those with children reported adjusting how they performed their role as fathers.

**Conclusions:**

Social context surrounding HIV diagnosis impact how heterosexual HIV positive men experience HIV related stigma and how they perceive themselves as men, which may influence their care seeking behaviors. These findings have implications for HIV programs geared towards African heterosexual men in general and HIV positive men in particular.

## Background

Efforts to address the HIV/AIDS epidemic has generally focus on women because they account for over 50 % of people living with HIV/AIDS (PLWHA) globally [1]. According to the UNAIDS Global AIDS Epidemic Report, 80 % of women aged 15 and older living with HIV are in sub-Saharan Africa, despite the declines in new infections of the disease [1]. These have been attributed to several factors such as gender roles, socio-cultural expectations, power dynamics in relationships, and physiological vulnerability that predispose women than men [2–4]. Most work on HIV-related stigma has focused on women’s experiences of stigma and has described such experiences as being more intense than those suffered by men [3, 4]. However as Lynch and colleagues note, “focusing on the position of women in the [HIV] epidemic has led to overlooking the needs of men living with the disease” [5], specifically heterosexual HIV-positive men.

Wyrod argues that heterosexual HIV positive men are also victims of issues faced by women living with HIV/AIDS [6]. However, there is limited research on how HIV positive heterosexual men live with HIV/AIDS, or experience HIV-related stigma. This is especially the case in Sub-Saharan Africa where the epidemic is highest and where men account for over ten million of PLWHA (that is over 50 % of men living with HIV/AIDS globally) [1]. Few preventive efforts have attempted to engage heterosexual African men, although the “main assumptions behind HIV policies in many parts of [Africa] is that heterosexual practices play a major role in the spread of the virus” [7]. This singular focus on women’s experiences with HIV/AIDS or HIV-related stigma in Sub-Saharan Africa has created a backlash of what Stillwagon describes as a perpetuation of the stereotype of a hyper-sexualized African male spreading HIV [8]. This, she argues, has oversimplified the construction of masculinity, while Lynch, Brouard and Visser note that it has “contributed to the development of a theme of female oppression in discussions about HIV and AIDS, with very little critical exploration of men’s subjectivities” [5]. The consequence is a downplaying of African heterosexual men’s experiences of the disease. The few studies that have explored heterosexual HIV positive African men’s subjectivities of living with HIV/AIDS have taken place in southern Africa [5, 6, 9–11] or among African-born men who live in the diaspora [7]. There are even fewer studies in West Africa that have explored the lived experiences of HIV positive African heterosexual men, and most have focused on the reproductive desires of men and women living with HIV/AIDS [12–16]. In this article, we attempt to address this gap by exploring the situation among heterosexual HIV positive men in southwest Nigeria.

Nigeria, the most populous country in Africa, is listed as one of the countries with the largest HIV/AIDS epidemic in Sub-Saharan Africa that is showing signs of stabilizing or declining [1, 17]. With 3.4 million people living with HIV/AIDS (PLWHA), the country currently has the second highest absolute number of people living with the disease globally, after South Africa [1]. Similar to other Sub-Saharan African countries, HIV prevalence is higher among women (3.5 %) than men (3.2 %) [17]. While the provision of antiretroviral therapy (ART) has improved the lives of HIV patients in Nigeria, the fear of unwittingly disclosing their HIV status and the resulting stigma have been identified as two of the factors that impact HIV patients’ lives [18, 19]. However, exactly how heterosexual HIV positive Nigerian men experienced these and other factors are yet to be explored within the purview of the cultural construction of masculinity.

Wyrod’s work on masculinity and AIDS stigma provides the conceptual framework for this study [6]. Drawing from the conception of stigma as a social process and gender as a social construction, Wyrod argues that heterosexual HIV positive men fail to “embody hegemonic notions of being a man [which] requires men to deny weakness and vulnerability… and be self-reliant family providers” [6]. Since there are multiple forms of masculinity in any setting, this study uses Wyrod’s description of hegemonic masculinity as “the dominant form of masculinity in a given social context” [6]. This form is the prevailing “cultural model of the idealized manhood” and “it is the frame used by individual men to judge their “success” as men” [20]. Though there is no one construction of African or Nigerian masculinity, research has shown that a primary mandate or chief requirement for manhood in Africa is “achieving some level of financial independence, employment or income, and subsequently starting a family” [21, 22]. Wyrod argues that HIV/AIDS undermines this mandate for African heterosexual HIV positive men because many equate “being HIV positive with no longer having intimate relationships, being unable to produce children and not being able to earn money to provide for their families – all key components of the hegemonic masculine ideal of being a provider [man]” [6].

As such, heterosexual HIV positive men experience stigma on a three-part dimensional schema: intrapersonal, interpersonal and structural. Intrapersonal stigma also known as internalized or self-stigma is internalized feelings of shame or blame based on negative social judgments of one’s identity. This can lead to anticipated stigma, which is defined as the reactions that an HIV positive person expects from others if it were known that he/she was HIV positive. Interpersonal dimension of stigma, also known as external or enacted stigma is actual acts or what people do to unfairly discriminate against a known or suspected HIV positive person [23]. The structural dimension of stigma is experienced when the above “micro-level processes work to reinforce the [hegemonic] gender order that subordinates HIV positive men and undergirds gender inequality at a social structural level” [6]. For this study, we explored the intra-and interpersonal stigma experiences of heterosexual HIV positive men in southwest Nigeria. Similar to Doyal and colleagues, this study focuses on the men’s lived experiences of HIV/AIDS and its stigma, and not on their roles as partners of women unlike other research in this area [7].

## Methods

### Study setting and participants recruitment

Data collection took place in Ibadan and Eruwa, in southwest Nigeria. Ibadan is one of the largest metropolitan cities in southwest Nigeria, with an estimated population of three million people [24, 25]. Ibadan is the administrative capital of Oyo state, and is home to a number of companies, businesses, and higher institutions of learning. With HIV/AIDS prevalence rate of 5.6 % in Oyo state [17], six of the nine HIV/AIDS treatment centers in Oyo state are based in Ibadan, along with a number of HIV/AIDS response activities. Eruwa is a small rural town located about 38 miles outside of Ibadan in the Ibarapa East Local Government Area (LGA) of Oyo state [26]. Ibarapa East LGA has an estimated population of 134,101 [27], shared between Eruwa, Lanlate and other towns. The inhabitants are subsistent farmers and petty traders. The decision to collect data in these two settings was based on prior fieldwork and existing collaboration with community based organization (CBO) that provides services in these areas. Under the directorship of a physician, four staffs and five volunteers, the CBO works to provide HIV/AIDS prevention, care, and support services in underserved and unreached communities. It reaches out to women at maternity centers, churches and through birth attendants for HIV counseling and testing, support for ART and follow**-**up. The CBO also provides mobile- and facility-based HIV counseling and testing services; assists HIV positive individuals to access treatment by referral to the urban-based antiretroviral clinics through its Rural AIDS Care, Support and Treatment Access (RACTA) program; and facilitates the formation of peer support groups especially for the underserved and isolated PLWHA in the rural areas. Participants were recruited through community-based organization.

Participants were recruited using purposive sampling of HIV positive patients [28]. Each community has an established HIV support group that meets bi-weekly. The CBO provided information about the study to peer leaders of the support groups in both study settings. Each peer leader then contacted their members about the study. As agreed upon by members in these support groups, focus group discussions and in-depth interviews were scheduled to coincide with their support meetings. Before starting the discussions and interviews, we informed participants about the purpose of the study, and advised that participation was completely voluntary. The criteria for participation were 18 years or older and clinical diagnosis of HIV sero-positive status. Consenting self-identified HIV positive participants either signed or thumb-printed the consent forms. Participants then completed (either by self or through the assistance of a facilitator) a survey on gender, age, education, ethnic group, religion, employment, marital status, number of children, clinical diagnosis of HIV, and length of time since HIV diagnosis. Community-based organization’s staffs confirmed clinical diagnoses of participants’ HIV status.

### Data collection

Data was collected using in-depth interviews, focus group discussions, and field journals. Combining these approaches allowed participants to express their views on a range of issues in the most comfortable context for them. Field journals were kept during focus groups and interviews for continuous reflexivity [29]. Following the completion of the consent forms and surveys, participants were asked individually whether they want to be part of a group discussion or meet for one-on-one in-depth interview. All agreed to the focus group discussion with the exception of those who came late or indicated that they had other appointments and may have had to leave before the end of the group discussion. Focus groups and in-depth interviews were held at the same location used for support group meetings. As confirmed by peer leaders, all members of the HIV support group in each community came for the study, and only participants and research team were present during data collection.

Discussions and interviews were conducted in at three languages: English, Pidgin English and Yoruba. This was to enable participants’ to express themselves in the language(s) with which they were most comfortable and to allow the use of local words that could not be translated into English, or words with meanings that may have become lost in translation. Interviews conducted in Ibadan used all languages, while interviews conducted in Eruwa used mainly Yoruba, with occasional English words. Without using the word “stigma,” interview and discussions were guided by four questions: What was your reaction to your HIV diagnosis? What challenges do you experience in living with HIV/AIDS? What changes have you made (both positive and negative) since your HIV diagnosis? How do your family, friends and community members react to your HIV status? The word stigma was only used after it was introduced by any of the participants. Development of discussion questions was informed by literature review on lived experiences of PLWHAs, research team’s experience of working with PLWHA, and suggested research questions and methodologies by Deacon [23]. The discussion guide was translated into Yoruba and Pidgin English and back translated to ensure consistency and verify content.

A male facilitator, a member of the research team, with over five years of experience working with PLWHA in the community and fluent in the three languages, conducted the interviews and focus groups. This was done in recognition of the cultural context of gender dynamics in the study settings and to allow for open discussion among participants. Though participants were encouraged to use pseudonyms during discussions, all introduced themselves by their own names, as they said, “*gbogbo wa la mo ara wa*” (meaning: we all know each other). During focus groups especially, probes were used to elicit more responses or expand ideas expressed by participants [30]. Five in-depth interviews and two focus groups discussions were conducted: three interviews were conducted in Eruwa, and two interviews were conducted in Ibadan. One focus group was conducted in each community, with four participants in Eruwa, and eight participants in Ibadan. Each group met once, with discussion lasting for an average of two hours, while in-depth interviews lasted for 45 min. The facilitator provided summaries of the discussions and interviews at the end of each meeting to allow for confirmation and clarification by participants. Participants were given monetary incentives to compensate for their time and transportation costs. All discussions and interviews were audiotaped and transcribed, first into the language in which the discussion or interview was conducted, and then into English. All names were removed from transcripts. Transcripts were loaded into the NVivo 8.0 software package for qualitative data management to facilitate data analysis.

### Data analysis

Data was analyzed using thematic analysis guided by social constructivist thinking. Social constructivist thinking postulate that individuals construct concepts, models and schemes to make sense of their experiences, and continually modify these constructions in light of new experiences [31]. These subjective meanings of experiences “are negotiated socially and historically, and formed through interaction with others, historical and cultural norms that operate in individual’s lives” [32]. This approach was chosen because stigma is a social process in the same way gender is a social construction. Both are constantly negotiated and re-evaluated in light of new experiences, such as HIV/AIDS. Construction of meanings and experiences can occur at the individual level or at the group level where people develop meanings for their activities together [32]. Examining HIV lived experiences is necessary at both levels in order to explore consensus or dissonance in individual or shared meanings among participants.

Braun and Clarke’s guide on conducting thematic analysis was used in the reading and coding of the transcripts [33]. Prior to coding, transcripts were read and re-read to allow for complete immersion in the data, identify possible patterns and drive the inductive process. Transcripts were then coded using open coding, such that each unit of text was assigned a code(s). All transcripts were coded by two members of the research team and corroborated by a third. Codes were reviewed and evaluated to ensure agreement among coders and discuss any discrepancy. The initially generated number of codes was 23. These were then collated for specific themes using axial coding by reorganizing and making connections between categories and subcategories, generating five thematic categories. Further review and evaluation led to three final thematic categories and delineation of the social context from categorical themes. An exhaustive set of data to support each thematic category was identified. Interpretation of meanings was guided by review of literature on lived experiences of HIV patients, authors’ experience in working with PLWHA (an average of fifteen years), authors’ shared cultural norms with participants, and detailed observational field journals. To ensure coherence and maximize rigor, community-based organization staffs and an independent researcher reviewed and validated the interpretations.

## Results

A total of 17 men participated in the in-depth interviews and focus group discussions (10 in Ibadan, 7 in Eruwa). Participants’ ages range from 38 to 53 in Ibadan, and 30 to 57 in Eruwa. From the time of clinical diagnosis, participants in Ibadan had lived with HIV/AIDS for an average of 4.85 years, while those in Eruwa reported an average of 1.66 years. All participants reported they were married with children, and over half (14) reported being unemployed. All participants in Ibadan had some level of education, while only one participant in Eruwa reported receiving any formal education. In addition, all were receiving antiretroviral therapy through the Presidential Emergency Plan for AIDS Relief program (PEPFAR), and were asymptomatic at the time of data collection.

While all the participants discussed a wide range of responses to their illness and their experiences in living with the constraints of HIV/AIDS, analysis of their responses revealed three major themes: 1) reaction to HIV status; 2) HIV stigma experience; and 3) lifestyle changes due to HIV positive status. Two of the above themes, 1) reaction to HIV status, and 2) HIV stigma experience are interrelated by the social context surrounding HIV diagnosis. Using Pasick and colleagues’ description, social context is defined as “the sociocultural forces that shape people’s day-to-day experiences and that directly and indirectly affect health and behaviors” [34]. Social context surrounding HIV diagnosis serves as the milieu in how the men described their illness and how they responded to it.

### Social context surrounding HIV diagnosis and reaction to HIV status

Social context surrounding the HIV diagnosis of participants influenced their reactions to their HIV diagnosis. These factors are divided into two: when HIV diagnosis is preceded by participant’s own illness (symptomatic at diagnosis), or when HIV diagnosis is preceded by HIV diagnosis of an immediate family member (participant is asymptomatic at diagnosis). When HIV diagnosis was preceded by months of a participant’s own sickness, participants reported surprise or shock at their diagnosis:

As a man, you are not supposed to get tired, at least not all the time. But I discovered I use to be very tired from the inside, all the time and I couldn’t work. My tongue use to be very white and I became very skinny. I eventually went for a medical test and the doctor told me that I am infected with HIV virus. I was shocked when I heard because my wife and children tested negative but I was the only one that was positive. (Eruwa FG)

I was learning a trade, but my sickness of malaria all the time prevented me from continuing. I was stooling a lot, and wasn’t sure what was wrong. My boss said to me, “what’s with this sickness of yours?” He advised me to go to the hospital…when I went back to the hospital I was told I have HIV. I was shocked. I don’t know how I got it. I did not eat for days. (Eruwa FG)

It was a real shock because my mother thought I was impotent [when asked why his mom thought he was impotent, he answered]: she’s never seen me with any woman…until I got married. My wife was a virgin when we got married. I was the one that dis-virgined my wife. I thought of committing suicide because we both trusted each other. (Ibadan FG)

When I was sick, I went to the hospital and they treated me for malaria, but the sickness persisted. I was asked to do a test, and it was through this test that I got to know about it…I was sad when I first heard about my status. I even refuse to listen to the news from my radio because I thought I will die soonest. (Eruwa FG)

It is of note that participants who were symptomatic at the time of diagnosis were more likely to use phrases like “as a man…” in explaining their responses. Participant’s comment that “as a man you are not suppose to get tired all the time” and the reference to sexual impotence is a reflection of the prevailing cultural construction of masculinity [22]. Baker and Richardo report that there is a social expectation and sanction that “a man should have sexual relations with a number of women by the time he gets married” [21]. Orobaton reports similar findings among young men in southwest Nigeria, and that lack of sexual conquests may lead to scorn [35]. As such, one participant’s comment about his mother’s thoughts of his sexual virility is in line with this expectation. As shown below, participants who reported shock also admitted contemplating suicide, had experienced concern about how others would react to their diagnosis, and worried about the potential cost of treatment:

I was sad about it, because I did not really know how I got it. I thought about killing myself. I moved closer to God. (Eruwa In-depth interview)

When I was told, I was afraid because my parents are poor. I was afraid because of the money I will need to treat myself, even transportation expenses would be an issue. (Eruwa FG)

It really shocked me … I was surprised about it because I am a clergyman. What will people say? …Over some weeks I was not myself. (Ibadan FG)

At least, two participants reported the loss of their spouses, who either left them or were taken away by their relatives:

When I was in Calabar, I use to be very sick, and I was working a lot. I was asked to go to the hospital for a bed rest… but you know as a man, I just continue working. After a while, I went to the hospital … It was the doctor that did the test that told me my HIV status that I am positive. When I informed my wife she said she would sue for divorce…I said to her, why sue for divorce? (Ibadan FG) [Participant did not confirm whether or not his wife went through with the divorce]

My wife left me alone and till now I am still single because she refuses to return to me. My brothers heard about it and did not react negatively to me. (Ibadan FG)

“…My wife was taken away by her family members…” (Ibadan in-depth interview)

The loss of their wives is also a loss of their place on the existing hegemonic ideal of being a man, which requires ‘real men’ to have wives [22], and a woman leaving a man, especially if they don’t have children exposes the man to ridicule [35]. It is of note that all participants indicated that they were married with children on the demographic survey, and none indicated that they were separated or divorced. The details about their marital status only emerged during the group discussions or interviews. It is postulated that this may be because of the belief that once couples have children together, they have a bond for life since children establish the blood connection between couples thereby “secur[ing] conjugal ties” and cementing the relationship for life [36].

On the other hand, when their HIV diagnosis was preceded by that of a family member (e.g., wife or son), participants reported not being shocked or surprised, and were accepting of the news:

Through my wife, when she got pregnant and I went for the HIV confirmatory result with her. It was then that I was told that I would need to do a blood test. Then I got to know about my HIV status. It was through my wife pregnancy… I was not surprise or amazed at all about my status because we have been enlighten through the television media. (Ibadan FG)

I got to know through my son. We went to hospital called “Oni and Son.” He was tested positive and when I was tested too it was discover that I was infected… I was not too afraid because it is not a disease that will kill in a speed of light. (Ibadan FG)

I got to know through my wife when she was sick, and we took her to the hospital, so they said she had the HIV virus but that I was negative, that I should report back for a new test six months later. It was after this window period I was tested positive…I was not surprised. (Eruwa FG)

As seen from the above quotes, participants were not surprised and offered explanations as to why they were not – education through media or awareness that “*it’s not a disease that can kill in a speed of light.*” This also indicates a measure of acceptance on their part about their status. It is possible that this acceptance maybe due in part to the fact that following a family member’s diagnosis, participants may have suspected they had HIV and had prepared themselves by seeking information about HIV/AIDS. In fact, participants who reported finding out about their HIV status through the diagnosis of a family member indicated they would not have otherwise known because they were not sick or ill in any way, that is, they were asymptomatic at the time of diagnosis. Four participants in Ibadan reported knowing about their HIV status following the diagnosis of an immediate family member, while two reported the same in Eruwa. In addition, participants whose diagnosis was preceded by that of a family member reported educating others about HIV/AIDS, and receiving support from family members:

I try to enlighten people about it. (Ibadan FG)

I encourage people that are victims of the virus and I have directed about four people to the teaching hospital for treatment and medication. I try to get close to people that I perceived are in that condition. (Eruwa FG)

It is only my family members that know but they don’t tend to neglect me but instead they supported me financially and materially. (Ibadan FG)

Participants become advocates by educating others about HIV/AIDS. Their reports of family support may be due to the fact that the knowledge about their status may have become ‘known’ or suspected by other family members with the diagnosis of an immediate family member, especially wife or child, and the HIV test was merely a confirmation. It is postulated that their acceptance may also be due to the fact that they have in some way met the burden of being a ‘man’: having a family, which confirms their virility, and not suffering an *apparent* physical illness, due to the asymptomatic phase of the disease. Whether participants were symptomatic or asymptomatic at the time of HIV diagnosis had an effect on their HIV stigma experience.

### Social context surrounding HIV diagnosis and HIV stigma experience

Most participants reported some experience of stigma. However, the intensity of the experience is impacted by how participants found out about their sero-positive status. Participants whose HIV diagnosis was preceded by their own illness reported experiencing enacted stigma from friends, family, and community members who avoided them:

People will remove their clothing where I spread mine because they don’t want to be infected… (Ibadan in-depth interview)

People were actually running away from me. They don’t even want to sit close to me at all…if I give a gift to their children they will throw such gift away. (Eruwa FG)

Many of my friends refuse to come near me when they heard. The way the government is talking about AIDS has created a lot of fear in the heart of the masses. (Ibadan FG)

They also reported self-imposed isolation in response to anticipated stigma:

I use to have many friends but when I discover my status I begin to separate myself from many of my friends because I don’t want them to know my status. (Ibadan FG)

One day I was at home, some ladies were talking about HIV. It was as if they know I was infected. So I left the vicinity. (Ibadan FG)

It is possible that the persistence of the men’s sickness along with their physical appearance influenced how family, friends and community members responded to them. Keeping in mind that men are expected to be strong, and healthy [21], being sick as reported by these men does not fit into the community construction of masculinity. As such, they experience stigma as compared to the men who reported finding out about their status through their immediate family’s diagnosis:

There is no change. The way I relate to my family is still the way I relate to them before [HIV]. (Ibadan FG)

It is only my family members that know but they don’t tend to neglect me but instead support me financially and materially. (Ibadan FG)

I did not really see any much change. I still spend time with my friends and family. (Eruwa In-depth)

As seen above, men whose diagnosis was preceded by that of a family member did not seem to be subjected to enacted stigma experiences. It is suggested that this may be because they were yet to experience the illness associated with the disease and, as such, still fit into the ‘healthy masculine image’ [5]. By the same token, it should be noted that only their family members know about their HIV status, and none of them reported informing anyone outside of their family about their status. It is possible that their experience about stigma may change if others besides family members are aware, or if the men become symptomatic. As research has demonstrated, HIV seropositive status impact lives and demand that patients make changes.

### Lifestyle changes due to HIV positive status

All the participants reported making changes to their lifestyles, especially with respect to sexual intimacy. Some participants talked about reducing sexual intercourse with their wives or engaging in extramarital affairs, while others reported condom use for sexual activity. In addition, many talked about reducing their fun-loving ways of drinking and “gyrating” (colloquial meaning: going to clubs):

I have stop womanizing, I now use condom. I have reduced my sexual interaction with my wife because I don’t have so much strength. (Eruwa FG)

I stop having sex with other women and I make use of condom when I want to have sex with my wife. (Eruwa FG)

I use to gyrate a lot and drink beer before but now I stopped it when I heard about my status. (Ibadan FG)

Others talked about instructing their children not to use their personal belongings like razor blades, while others reported not using their teeth to share meat with their children:

… the tooth brush I use to share with my children at home I no longer do that again. I don’t even use my teeth to share meat for my children again. Also the [razor] blade I use for my nails I don’t allow my children to use it. (Ibadan FG)

I placed my [razor] blade at a place where the children can’t reach it. I also told them never to touch it. (Eruwa FG)

The practice of using ‘teeth’ for sharing meat, though fading out, is still practiced by some in this context. There is a cultural expectation of fathers sharing food with their children, as the situation provides them with the opportunity to socialize with their children. In fact, a sign of fatherhood, a central piece in the construction of masculinity in this context, is having your children gather around you while you eat so you can share food with them and teach them social mores [37]. The change in this social context is that HIV positive men have to adjust how they engage in this behavior. Such changes, though insignificant on the surface, have a bearing on how the men currently see themselves and the constraints that HIV impose on their lives.

## Discussion

This study explored the lived experiences of heterosexual HIV positive men in southwest Nigeria. Specifically, we examined how the social context surrounding HIV diagnosis impacted reaction to the diagnosis and stigma experience within the cultural context of how masculinity is constructed. The data suggest that when HIV diagnosis is preceded by one’s own experience of illness, participants were likely to report shock and surprise at their diagnosis and be less accepting of their HIV status as opposed to when HIV diagnosis is preceded by diagnosis of immediate family members. Reaction of shock and fear at diagnosis has also been reported from research among African heterosexual HIV positive men although these studies were not exploring the context surrounding HIV diagnosis [5–7]. In their study of heterosexual African men living with HIV in London, Doyal and colleagues reported that the shock of their HIV diagnosis left participants in their study feeling that their “future had been taken away from them,” while others reported attempted suicide by heterosexual HIV positive men in South Africa and Uganda following HIV diagnosis [5, 6].

Furthermore, men whose persistent illness preceded their diagnosis experienced enacted stigma from friends, family, and community members. This is in line with the argument of Lynch and colleagues that it is the experience of HIV related illness in particular that disrupts the “normative expectations of men to be invulnerable and self-reliant.” [5]. Colvin and colleagues posited that the experience of being profoundly ill challenges HIV positive men’s sense of self and sparks a critical reflection of their identities [38]. These men, therefore, experience isolation and avoidance from community members because they do not fit the cultural construction of masculinity and have a difficult time accepting their HIV positive status. Such masculine constructions, especially as practiced in patriarchal societies, of which many of the ethnic groups in southwest Nigeria engage in, is described as being heteronormative [20], and require men to validate their gender by achieving prescribed standards of maleness, as such being strong, stoic, with no room for weakness or sickness. Men in this study who were sick before their diagnosis failed to achieve these standards, and as such, they had an intense experience of isolation and stigma. Contrarily, men whose diagnosis was preceded by that of a family member were able to maintain the “notion of the healthy, invulnerable male” [5], and were accepting of their status, with some reporting educating others about HIV/AIDS. Acceptance of HIV positive status has been shown to be necessary for behavior change [39] and medication adherence [40].

Research has explored the issue of parenthood for PLWHA especially African women in their construction of motherhood in the context of HIV/AIDS [41, 42]. Lately, though limited, research has focused on examining the experience of fatherhood by HIV positive African heterosexual men [7, 21]. Drawing from the words of a young HIV positive father in Baker and Richado work in South Africa, “If …you are HIV positive, like many of us, that is the worst state that a man can be in,” it is obvious that being an HIV positive father presents challenges of renegotiating the landscape of dispensing fatherly responsibilities [21]. Comments by participants in this study indicate some adjustments made by HIV positive fathers that though insignificant on the surface, are necessary for them to maintain some semblance of being men and, especially, of being fathers if they are to live up to the local ideals of masculinity and fatherhood. Cultural expectations of fathers socializing their children using specific cultural situations such as food sharing had to be readjusted. However, unlike findings from Doyal and colleagues [7], none of the participants in this study reported loss of a relationship with their children. This may be because they are ‘home’ in their own communities where the prevailing belief is that “the child will always know the father” (*omó ma mo bàbá è*) as such “you can never take children away from their father” (*kò sí eni tó le gba omo lówó olómo*). In addition, it is postulated that the close-knit communities (especially in Eruwa) in which the men live provides them with access to their children and the opportunity to carry out their role as fathers. More research is needed to further explore how community setting influences the performance of HIV positive fathers’ and the strategies that they use to meet the cultural expectations of being fathers.

The results from this study contribute to the growing research that men can change their sexual behaviors in response to HIV/AIDS, and challenge the stereotype of the hypersexualized African male spreading HIV [5, 7, 8]. Similar to other findings reviewed, participants reported changing their sexual behaviors by engaging in safer sex and reducing both alcohol use and extramarital affairs [7]. Mfcane posits that heterosexual HIV positive African men are redefining and reconstructing masculinity by refraining from “certain aspects of normative masculinity that are seen as jeopardizing their health” (cited in Lynch et al. [5]).

There are limitations in interpreting the results from this study. The sample was small and geographically limited to heterosexual HIV positive men who were members of support networks in Ibadan and Eruwa, in Oyo state. As such, the views and beliefs expressed by participants cannot be generalized to other heterosexual HIV positive men in other communities. In addition, the interpretative lenses used in exploring the men’s experiences are based on the cultural norms of the Yoruba people in southwest Nigeria, and may not necessarily translate to other cultural groups. Despite these limitations, findings from the study contribute to knowledge on how the social context surrounding HIV diagnosis of heterosexual HIV positive men influences reaction, experience of stigma, and construction of masculinity.

## Conclusions

By exploring the social context surrounding HIV diagnosis, the current study provides insight into how factors leading to African heterosexual men’s HIV diagnosis can enable or hinder their acceptance of their status, which has a bearing on how they see themselves as men, and potentially how they seek care and support. HIV/AIDS programs geared toward men can benefit from such insights by framing testing for HIV *now* rather than later when illness is experienced, as a strategy for preventing exposure to suspicion and possible stigma from family, friends and community members.

## Abbreviations

ART, Antiretroviral Therapy; CBO, Community Based Organization; HIV/AIDS, Human Immunodeficiency Virus/Acquired Immunodeficiency Syndrome; LGA, Local Government Area; PEPFAR Presidential Emergency Plan for AIDS Relief; PLWHA, People Living With HIV/AIDS; RACTA, Rural AIDS Care, Support and Treatment Access.

## References

[1] UNAIDS Global AIDS Epidemic Report, UNAIDS. Geneva, Switzerland: Joint United Nations Programme on HIV/AIDS; 2015.

[2] Goldstein N, Pretorius HG, Stuart AD (2003). The social construction of HIV/AIDS. Health SA Gesondheid.

[3] Nepal B (2007). Prosperity, equity, good governance and good health: focus on HIV/AIDS pandemic and its feminization. World Health Popul.

[4] Quinn TC, Overbaugh J (2005). HIV/AIDS in Women: An Expanding Epidemic. Science.

[5] Lynch I, Brouard PW, Visser MJ (2010). Constructions of masculinity among a group of South African men living with HIV/AIDS: reflections on resistance and change. Cult Health Sex.

[6] Wyrod R (2011). Masculinity and the persistence of AIDS stigma. Cult Health Sex.

[7] Doyal L, Anderson J, Paparini S (2009). “You are not yourself”: exploring masculinities among heterosexual African men living in London. Soc Sci Med..

[8] Stillwaggon E (2003). Racial metaphors: interpreting sex and AIDS in Africa. Dev Change.

[9] Sorrell JBJ, Rafaelli M (2005). An exploratory study of constructions of masculinity, sexuality and HIV/AIDS in Namibia. Cult Health Sex.

[10] Shamos S, Hartwig KA, Zindela N (2009). Men’s and women’s experiences with HIV and stigma in Swaziland. Qual Health Res.

[11] Skovdal M, Campbell C, Madanhire C, Mupambirey Z, Nyamukapa C, Gregson S (2011). Masculinity as a barrier to men’s use of HIV services in Zimbabwe. Global Health..

[12] Oladapo OT, Daniel OJ, Odusoga OL, Ayoola-Sotubo O (2005). Fertility desires and intentions of HIV-positive patients at a suburban specialist center. J Natl Med Assoc.

[13] Smith DJ, Mbakwem BC (2007). Life projects and therapeutic itineraries: marriage, fertility, and antiretroviral therapy in Nigeria. AIDS.

[14] Smith DJ, Mbakwem BC (2010). Antiretroviral therapy and reproductive life projects: mitigating the stigma of AIDS in Nigeria. Soc Sci Med..

[15] Chama C, Morrupa J, Gashau W (2007). Sex and reproduction among HIV-infected people in Maiduguri, Nigeria. Obstet Gynecol Int J.

[16] Iliyasu Z, Abubakar IS, Kabir M, Babashani M, Shuaib F, Aliyu MH (2009). Correlates of fertility intentions among HIV/AIDS patients in northern Nigeria. Afr J Reprod Health.

[17] National Agency for the Control of AIDS, Federal Republic of Nigeria. Global AIDS Response Country Progress Report. Abuja, Nigeria: Nigeria GARPR; 2015.

[18] Mohammed MD, Sarki R (2004). Adherence to antiretroviral drugs in North-Central zone of Nigeria. East Central Afric Pharma Sci.

[19] Olowookere SA, Fatiregun AA, Akinyemi JO, Bamgboye AE, Osagbemi GK (2008). Prevalence and determinants of nonadherence to highly active antiretroviral therapy among people living with HIV/AIDS in Ibadan, Nigeria. J Infect Dev Ctries.

[20] Jewkes R, Morell R (2010). Gender and sexuality: emerging perspectives from the heterosexual epidemic in South Africa and implications for HIV risk and prevention. J Int AIDS Soc..

[21] Barker G, Richardo C (2005). Young men and the construction of masculinity in Sub-Saharan Africa: implications for HIV/AIDS, conflict and violence. Social Development Papers: Conflict Prevention and Reconstruction.

[22] Olawoye JE, Omololu FO, Aderinto Y, Adeyefa I, Adeyemo D, Osotimehin B (2004). Social construction of manhood in Nigeria. Afr Popul Stud.

[23] Deacon H (2005). Understanding HIV/AIDS stigma. A theoretical and methodological analysis.

[24] Ajuwon AA, McFarland W, Hudes ES, Adedapo S, Okikiolu T, Lurie P (2002). HIV risk-related behavior, sexual coercion, and implications for prevention strategies among female apprentice tailors, Ibadan, Nigeria. AIDS Behav.

[25] Adedimeji AA, Omololu FO, Odutolu O (2007). HIV risk perception and constraints to protective behavior among young slum dwellers in Ibadan, Nigeria. J Health Popul Nutr.

[26] United States Agency for International Development. Nigeria HIV/AIDS Health Profile. Washington DC: USAID; 2010.

[27] Nigerian National Population Census. Abuja, Nigeria: National Bureau of Statistics of Nigeria; 2006.

[28] Patton MQ (2002). Qualitative Research and Evaluation Methods.

[29] Denzin NK, Lincoln YS (2011). The Sage Handbook on Qualitative Research.

[30] Morgan DL, Krueger RA (1998). The Focus Group Kit.

[31] Schwandt TA, Denzin NK, Lincoln YS (1998). Constructivist, interpretivist approaches to human inquiry. The Landscape of Qualitative Research: theories and Issues.

[32] Creswell JW (2003). Research Design: Qualitative, Quantitative and Mixed methods approaches.

[33] Braun V, Clarke V (2006). Using thematic analysis in psychology. Qual Res Psychol..

[34] Burke NJ, Joseph G, Pasick RJ, Barker JC (2009). Theorizing social context: rethinking behavioral theory. Health Educ Behav.

[35] Orobaton N. Dimensions of sexuality among Nigerian men: implications for fertility and reproductive health. In: Bledsoe C, Lemer S, Guyer J, editors. Fertility and the Male Life Cycle in the Era of Fertility Decline. New York: Oxford University Press. 2000.

[36] Dyer SJ (2007). The value of children in African countries – insights from studies on infertility. J Psychosom Obstet Gynaecol.

[37] Bascom W. The Yoruba of Southwestern Nigeria. Prospect Heights, Il: Prospect Heights, Illinois: Waveland Press; 1969.

[38] Colvin CJ, Robins S, Leavens J (2010). Grounding ‘responsibilisation talk’: masculinities, citizenship and HIV in Cape Town, South Africa. J Dev Stud.

[39] Liamputtong P, Haritavorn N, Kiatying-Angsulee N. Living Positively: The Experiences of Thai Women Living With HIV/AIDS in Central Thailand. Qual Health Res. 2011; doi: 10.1177/1049732311421680 10.1177/1049732311421680 21890710

[40] Okoror TA, Falade CO, Olorunlana A, Walker EM, Okareh OT (2013). Exploring the cultural context of HIV stigma on antiretroviral therapy adherence among people living with HIV/AIDS in southwest Nigeria. AIDS Patient Care STDS.

[41] Iwelunmor J, Zungu N, Airhihenbuwa CO (2010). Rethinking HIV/AIDS Disclosure Among Women Within the Context of Motherhood in South Africa. Am J Public Health.

[42] Okoror TA, Airhihenbuwa CO, Shisana O, Zungu-Dirwayi N, Smith E, Brown D, Louw J (2008). “My mother told me I must not cook anymore” - Food, Culture, and the Contexts of HIV and AIDS related Stigma in Three Communities in South Africa. Int Quart Comm Health Educ.

